# Long-term responses of Scots pine (*Pinus sylvestris* L.) and European beech (*Fagus sylvatica* L.) to the contamination of light soils with diesel oil

**DOI:** 10.1007/s11356-019-04328-6

**Published:** 2019-02-14

**Authors:** Agnieszka Bęś, Kazimierz Warmiński, Barbara Adomas

**Affiliations:** Department of Chemistry, Research Group of Environmental Toxicology, Faculty of Environmental Management and Agriculture, University of Warmia and Mazury in Olsztyn, ul. Prawocheńskiego 17, 10-720 Olsztyn, Poland

**Keywords:** Soil, Diesel oil, Scots pine, European beech, Morphological parameters, Chlorophyll fluorescence

## Abstract

Research into trees plays a very important role in evaluations of soil contamination with diesel oil. Trees are ideal for reclaiming contaminated soils because their large biomass renders them more resistant to higher concentrations of pollutants. In the literature, there is a general scarcity of long-term studies performed on trees, in particular European beeches. The aim of this study was to evaluate the responses of Scots pines and European beeches grown for 8 years on soil contaminated with diesel oil. Selected morphological and physiological parameters of trees were analyzed. The biomass yield of Scots pines was not significantly correlated with increasing concentrations of diesel oil, but it was more than 700% higher than in European beeches. Scots pines were taller and had a larger stem diameter than European beeches during the 8-year study. The diameter of trees grown on the most contaminated soil was reduced 1.5-fold in Scots pines and more than twofold in European beeches. The length of Scots pine needles from the most contaminated treatment decreased by 50% relative to control needles. The shortest needles were heaviest. The fluctuating asymmetry (FA) of needle length was highest in Scots pines grown on the most contaminated soil, whereas the reverse was noted in the FA of needle weight. Diesel oil decreased the concentrations of chlorophylls *a* and *b*, total chlorophyll, and carotenoids. The Fv/Fm ratio of needles and leaves was influenced by the tested concentrations of diesel oil. The results of the study indicate that the Scots pine better adapts (grows more rapidly and produces higher biomass) to long-term soil contamination with diesel oil than the European beech. In European beeches, growth inhibition and leaf discoloration (a decrease in chlorophyll content) were observed already after the first year of the experiment, which indicates that 1-year-old seedlings of European beech are robust bioindicators of soil contamination with diesel oil.

## Introduction

Environmental pollution with crude oil and petroleum derivatives is a widespread problem around the world. It is caused by oil rig and oil tanker disasters as well as the extraction, land transport, processing, and storage of fuels. The ecological consequences of petroleum contamination are observed in plants, animals, and humans in all ecosystems (Howarth 1989; Singer et al. 1990; Singh et al. 2004; Tamada et al. 2012; Klamerus-Iwan et al. 2015; Sagerup et al. 2016).

Petroleum substances are produced during the distillation of various crude oil fractions. These fractions can be divided into four main categories: light distillates (liquefied petroleum gas, gasoline, naphtha), middle distillates (naphtha, diesel oil), heavy distillates (heavy fuel oil, lubricating oils, paraffin wax), and remaining fractions (asphalt) (Mackerer et al. 2003). The global demand for petroleum continues to grow, and it increased to 95.0 million barrels per day between 2015 and 2016 (International Energy Agency 2016). Diesel oil is widely used in transportation. In 2016, the demand for diesel was highest in Europe and the USA and was considerably lower in Asia and Oceania at 4.70, 4.56, and 1.30 million barrels, respectively. According to World Oil Outlook 2011, the global demand for diesel oil will reach 11.3 million barrels per day in 2035. The predicted increase in demand has highly adverse implications for the natural environment. Diesel oil combines several petroleum fractions (paraffins, naphthenes, olefins, and aromatic hydrocarbons), and its chemical structure is responsible for its toxicity (Domínguez-Barroso et al. 2016). The toxicity of diesel oil is also determined by dose, time of exposure, and the exposed organism (Bona et al. 2011). The mean mortality rate of the freshwater crustacean *Daphnia magna* exposed to diesel oil has been determined at 78.34% and acute toxicity (LC50)—at 1.78 ppm. *Onchorynchus mykiss* is a more resistant organism, and its mortality rate under exposure to the lowest concentration of diesel oil (100 ppm) was determined at 38.33%, mean mortality after 96 h of exposure—at 70.71%, and acute toxicity (LC50)—at 133.52 ppm (Khan et al. 2007).

Soils are reclaimed with the use of higher plants, including selected varieties of poplars (*Populus* spp.), willows (*Salix* spp*.*), birches (*Betula neoalaskana*), white spruces (*Picea glauca*), balsam poplars (*Populus balsamifera*), and *Enterolobium contortisiliquum* which are characterized by high biomass and considerable resistance to high doses of polluting substances (Garbisu et al. 2002; Leewis et al. 2013). These plants should not only accumulate and degrade contaminants, but they should also be able to grow in degraded habitats. Trees are ideal for reclaiming contaminated soils because their large biomass renders them more resistant to higher concentrations of pollutants. Trees have extensive root systems which are capable of accumulating large quantities of polluting substances. Phytoremediation is a non-invasive, environmentally friendly, and cost-effective alternative to physical remediation methods. However, research into phytoremediation is still in its early stages and requires further long-term studies (Kamath et al. 2004).

Tree health is influenced by both biotic and abiotic stressors (Schaberg et al. 2008). The responses of trees to drought have been widely studied under both controlled and field conditions (Löf et al. 2005; Chakraborty et al. 2017; Gazol et al. 2018). The effect of soil contamination with heavy metals on plant biomass has also been extensively described in the literature (Derome and Saarsalmi 1999; Stefanowicz et al. 2016; Grobelak et al. 2017; Pająk et al. 2017). In contrast, there is a general scarcity of research into tree responses to soil contamination with petroleum derivatives (Leewis et al. 2013; Villacís et al. 2016).

Xenobiotics present in the soil environment inhibit plant metabolic processes such as the activity of oxidative enzymes (Hussain et al. 2018) and biogenic amines (Baciak et al. 2017); they disrupt photosynthesis and fluorescence emission (Sousa et al. 2014). Photosynthetic efficiency and biomass yield can be compromised even when the damage to the photosynthetic apparatus is not yet visible.

Diesel oil modifies the morphological and biochemical parameters of plants growing on contaminated soil. However, the responses of Scots pines and European beeches grown for 8 years on contaminated soil have never been investigated. There are no studies analyzing the influence of diesel oil on beeches, either. For this reason, this study was undertaken to describe the effects of diesel oil on Scots pines and European beeches and to determine which of the analyzed tree species is a better bioindicator of soil contamination with diesel oil and is capable of rapidly adapting to degraded habitats and producing large amounts of biomass.

The aim of this study was to evaluate selected morphological and physiological parameters of Scots pines and European beeches grown for 8 years on soil contaminated with diesel oil.

## Materials and methods

### Pot experiment

The experiment was conducted for 8 years (2009–2016) in the Experimental Station of the University of Warmia and Mazury in Olsztyn, Poland. Five 1-year-old seedlings of Scots pine and European beech were planted per pot. Each pot was filled with 14 kg of light soil contaminated with the following amounts of diesel oil (LOTOS Eurodiesel): 0, 3, 6, 12, and 24 g of diesel oil per 1 g of soil dry matter (Table 1). Soil moisture content was maintained at 75% field water capacity throughout the experiment. Soil moisture was monitored with the FOM/mts time-domain reflectometer (Institute of Agrophysics of the Polish Academy of Sciences, Lublin, Poland). The experiment was carried out in five replications (∑ = 50 pots). The pots were maintained under natural conditions.

**Table 1:** Selected chemical and physical properties of the study soil

Parameter	Measured value
pH_KCl_	7.11
pH_H2O_	7.72
P_2_O_5_ (mg/kg soil)	501.0
K_2_O (mg/kg soil)	175.0
Mg (mg/kg soil)	42.0
C (%)	1.84
N total (%)	0.141
Hydrolytic acidity (me/kg soil)	7.6
Granulometric composition (% fraction content)	
2–0.05 mm	72.21
0.05–0.02 mm	15.13
0.02–0.002 mm	11.30
< 0.002 mm	1.36

### Biometric parameters

Plant height was measured from the base of the stem to the tip of the main shoot after planting and in autumn of each year. Stem diameter was measured 1 cm above soil surface in the last year of the experiment. Ten pairs of 2-year-old needles were collected from every Scots pine before harvest. The length of each needle in the pair was measured to the nearest ± 1 μm by digital image analysis in the Image Tool 3.0 program (UTHSCSA, USA). The needles were dried to constant weight at a temperature of 60 °C for 48 h, and each needle in the pair was weighed separately.

### Fluctuating asymmetry of Scots pine needles

The developmental stability of Scots pine needles was determined based on fluctuating asymmetry (FA) as the difference in length in each needle pair, divided by the length of the longer needle. Two FA indicators were determined based on needle length (FAL) and needle weight (FAW) according to the following formulas (Kozlov et al. 2002):

$$ {\displaystyle \begin{array}{c}\mathrm{FAL}=\frac{\left(L1-L2\right)}{0.5\left(L1+L2\right)}\\ {}\mathrm{FAM}=\frac{\left(W1-W2\right)}{0.5\left(W1+W2\right)}\end{array}} $$

*L*1, *L*2: needle length
*W*1, *W*2: needle weight

### Chlorophyll content and phaeophytization quotient

The chlorophyll content of leaves and needles was determined spectrophotometrically in the first year of the experiment after xenobiotic application and in the last year of the experiment before biomass harvest. Plant material for analyses was prepared according to the procedure described by (Barnes et al. 1992). Ten leaves/needles were sampled from each plant. Each test tube was filled with 40 mg of ground leaves/needles and 4 cm^3^ of dimethyl sulfoxide (DMSO) for spectroscopy (Uvasol®, Merck, Germany). Test tubes were placed in a water bath with a temperature of 60 °C and extracted for 3 h in darkness. The solution was cooled to room temperature; 2 cm^3^ of the extract was diluted 1:1 with DMSO and allowed to cool to ambient temperature before diluting 1:1 with fresh DMSO. The absorbance spectrum (400 to 800 nm) was measured, and absorbance peaks were determined at 750.0, 648.0, 665.0, 480.0, 435.0, and 415.0 nm with the Hitachi U-1800 spectrophotometer (Tokyo, Japan). The concentrations of chlorophylls *a* and *b*, carotenoid content, and the phaeophytization quotient were calculated according to the following formulas (Chai et al. 2018):

$$ {\displaystyle \begin{array}{c}\mathrm{Chla}={C}_{\mathrm{Chla}}\cdot \frac{V_{\mathrm{e}}\cdot x}{m}\\ {}\mathrm{Chlb}={C}_{\mathrm{Chlb}}\cdot \frac{V_{\mathrm{e}}\cdot x}{m}\end{array}} $$

where

Chla, Chlb: chlorophyll concentration in plant material [μg·mg^−1^]
*V*_e_: volume of DMSO extract (*V*_e_ = 4 ml)
*x*: dilution coefficient
*m*: sample weight [mg]

$$ {\displaystyle \begin{array}{c}{C}_{\mathrm{Chla}}=12.47\cdot {A}_{665}-3.62\cdot {A}_{648}\\ {}{C}_{\mathrm{Chlb}}=25.06\cdot {A}_{648}-6.5\cdot {A}_{665}\end{array}} $$

where

*C*_Chla_: concentration of chlorophyll *a* in the extract [μg·ml^−1^]
*C*_Chlb_: concentration of chlorophyll *b* in the extract [μg·ml^−1^]
*A*_*i*_: solution absorbance at *i*th wavelength

$$ \mathrm{TCC}=\frac{\left[1000\bullet {A}_{480}-\left(1.29\bullet \mathrm{Chla}\right)-\left(53.78\bullet \mathrm{Chlb}\right)\right]}{220} $$

where

TCC: total carotenoid content
Chla, Chlb: chlorophyll content in plant material [μg·mg^−1^]
*A*_*i*_: solution absorbance at *i*th wavelength

The results were expressed in milligrams of chlorophyll per gram of leaf fresh matter.

$$ \mathrm{PQ}=\frac{A_{435}}{A_{415}} $$

where

PQ: phaeophytization quotient
*A*_435_, *A*_415_: solution absorbance at a given wavelength [nm]

### Greenness index of European beech leaves

The greenness index was determined in ten leaves from each plant with the use of the SPAD 502 chlorophyll meter (Konica Minolta, Japan). The measurements were performed only for European beech leaves due to morphological differences between the analyzed plants. The greenness index was expressed in unitless SPAD values.

### Chlorophyll *a* fluorescence

The maximum quantum yield of PSII (Fv/Fm) was measured in the needles/leaves growing in the top half of a new shoot (5 plants per pot, 2 measurements per plant) with the HandyPEA chlorophyll fluorescence system (Hansatech Instruments Ltd., UK). The measurements were repeated on the same plants throughout the experiment. The Fv/Fm ratio was calculated each year in August and at harvest. Leaves/needles were covered with clips for 30 min to inhibit the light dependent phase of photosynthesis. Fluorescence kinetics was induced on an area of 4 mm with light intensity of 3000 μmol m^−2^ s^−1^. Maximum fluorescence (F_M_) was measured for 100 μs. Kinematic data were analyzed in the Pocket PEA Plus V1.10 program (Hansatech Instruments Ltd.).

### Statistical analysis

The normal distribution of parameters in each independent group was determined by the Shapiro-Wilk test, and the residuals were also tested for normality. The homogeneity of variance was confirmed in Levene’s test. Factorial ANOVA (*F* test) was performed. The experimental factors were as follows: tree species, diesel oil dose, and year of study. Significant differences were determined in Tukey’s test at *p* < 0.05. Two-factor and three-factor analyses of variance and a regression analysis were carried out. The results of the experiment, including biomass yield, tree height, stem diameter, chlorophyll *a* and chlorophyll *b* content, chlorophyll *a* fluorescence, leaf greenness index (SPAD), and fluctuating asymmetry indicators, were processed in the Statistica 11.0 program (StatSoft Inc. 2017).

## Results

### Biometric parameters and biomass yield

**Fig. 1:**
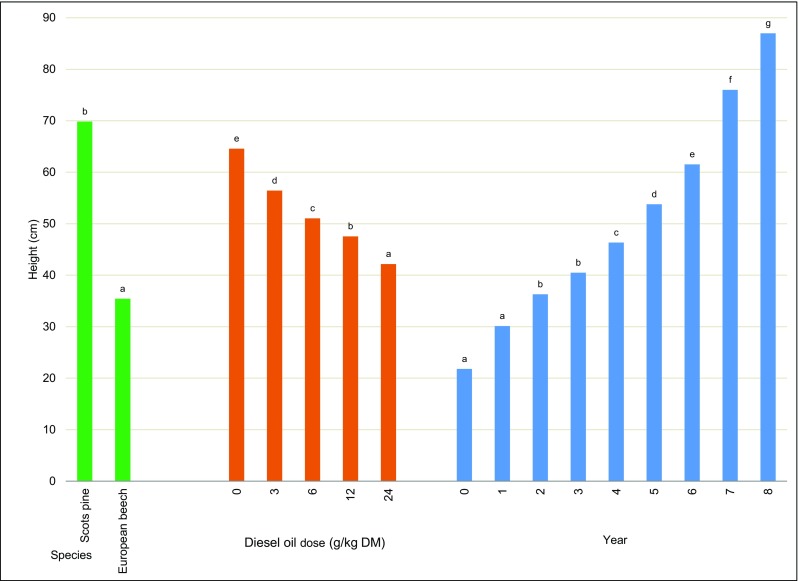
Average height depending on the tree species, the diesel oil dose, and the year of experiment. Different letters above the columns indicated significant difference at the *p <* 0.05

Diesel oil significant differentiated the biometric parameters of trees (Table 2). Scots pines were approximately 50% taller than European beeches. The tallest Scots pines and European beeches grew on control soil, whereas the shortest trees grew on soil contaminated with the highest dose of diesel oil (Fig. 1). The height of control trees and trees growing on contaminated soil increased by more than 74% during the 8-year experiment (Fig. 1).

**Table 2:** Analysis of variance (*F* test) of height of trees (Scots pine and European beech)

Source of variation	Sum of squares	df	Mean square	*F*	*p* value
Intercept	1,074,323	1	1,074,323	22,853.15	< 0.001
S	115,872	1	115,872	2464.85**	< 0.001
O	23,613	4	5903	125.57**	< 0.001
Y	116,246	7	16,607	353.26**	< 0.001
S × O	345	4	86	1.83n.s.	0.122
S × Y	47,988	7	6855	145.83**	< 0.001
O × Y	4370	28	156	3.32**	< 0.001
S × O × Y	1575	28	56	1.20n.s.	0.232
Error	13,727	292	47		

*S*, species; *O*, diesel oil dose; *Y*, year; *S* × *O*, *S* × *Y*, *O* × *Y*, *S* × *O* × *Y*, interactions between the factors

*Significant at *p* < 0.05; **significant at *p* < 0.01; *ns* not significant

**Fig. 2:**
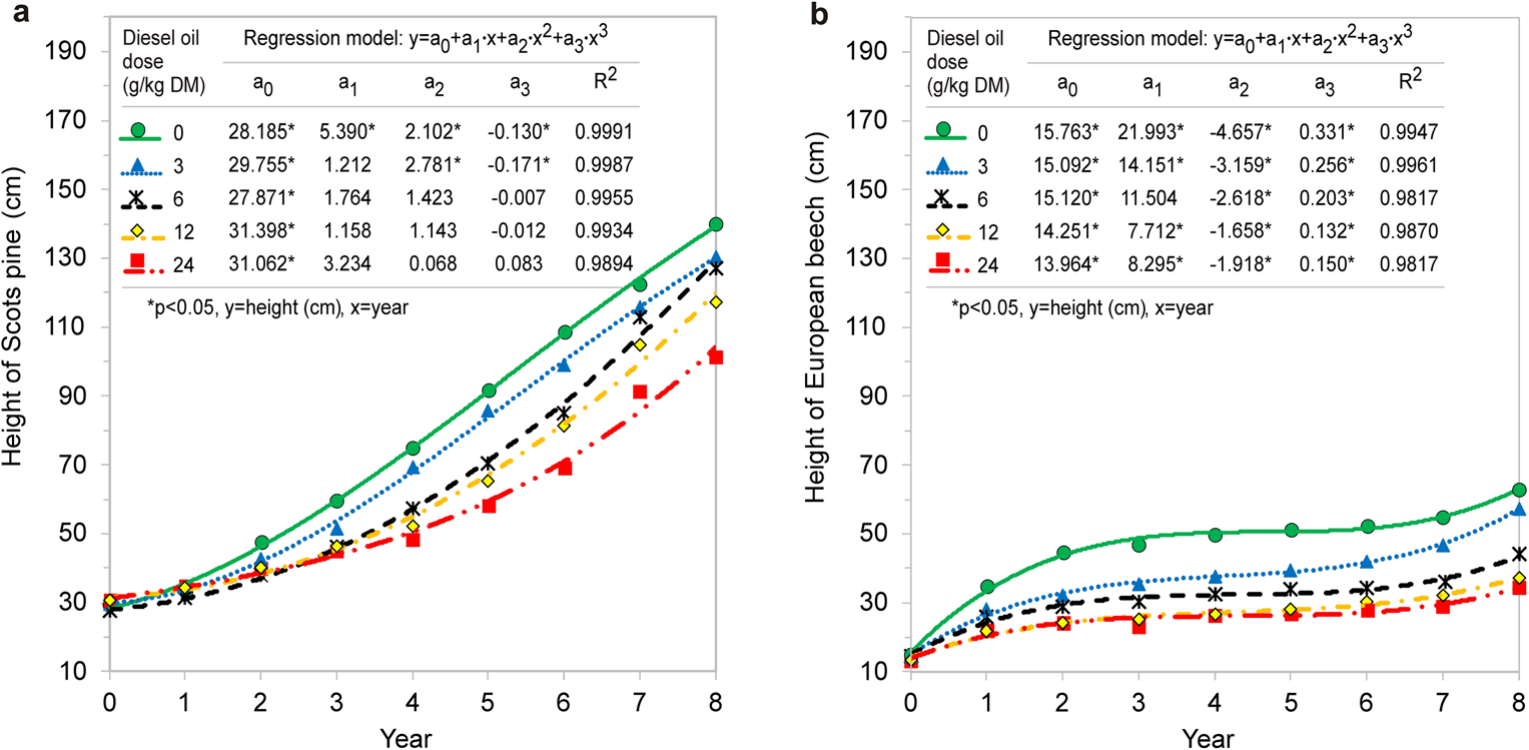
Temporary trends in the height of Scots pine (**a**) and European beech (**b**) depending on the diesel oil dose

**Fig. 3:**
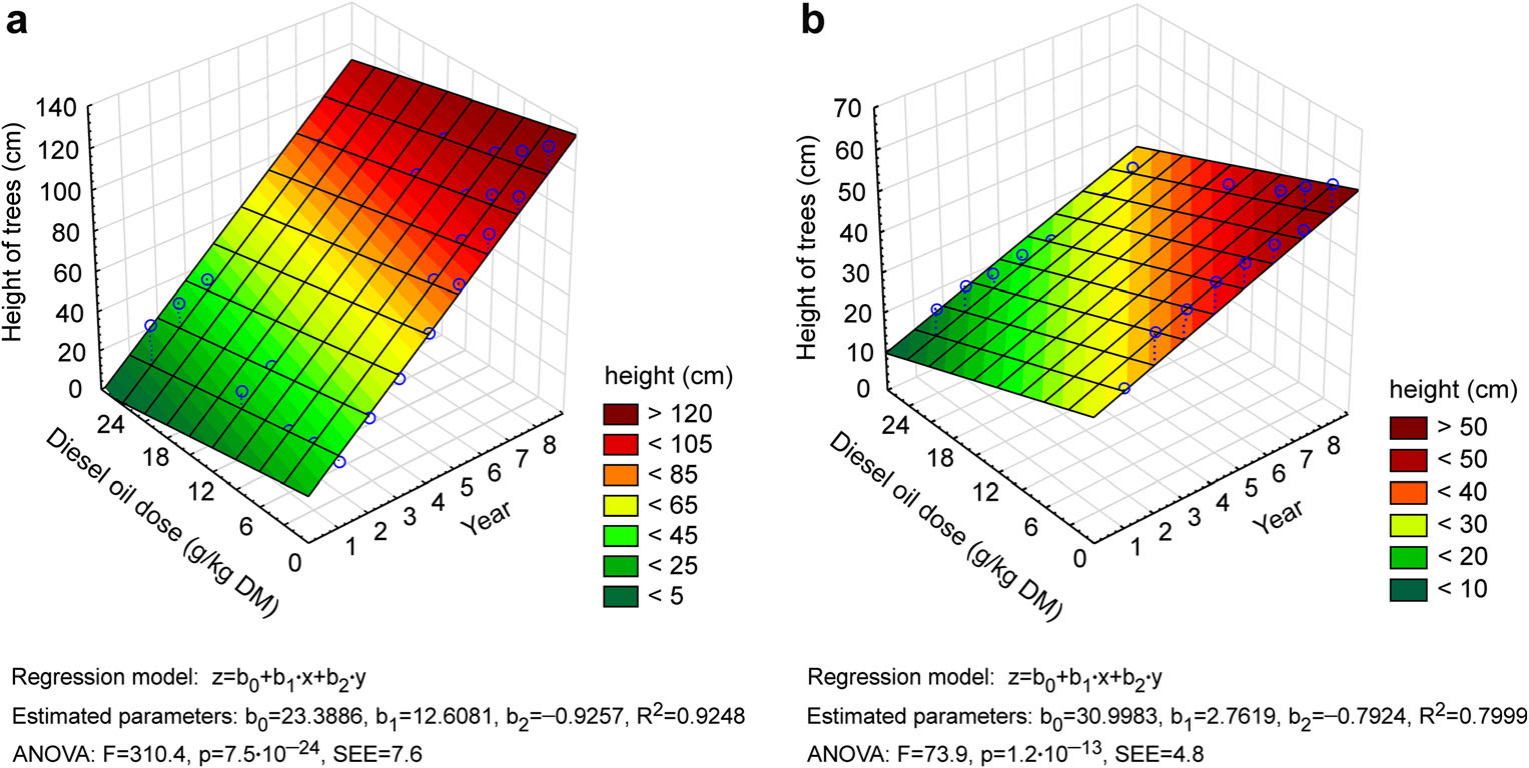
Relationship between the height of trees (**a** Scots pine, **b** European beech) and the diesel oil dose and the year of study. SEE is a standard error of estimation

In soil contaminated with diesel oil (12 and 24 g/kg soil DM), the growth of Scots pines was inhibited in the third year of the experiment. In contrast, the growth of European beeches on soil with the lowest dose of diesel oil was inhibited already in the first year. During the entire experiment, the height of Scots pines increased by 474% in the control treatment and by 322–442% in contaminated treatments. The height of European beeches increased by 405% in the control treatment and by only 271% in the most contaminated treatment (Fig. 2). The height of both tree species was significantly correlated with the experimental year. The quadratic function best fit the experimental data, and the value of *R*^2^ exceeded 0.98. All modeled parameters were statistically significant for the height of European beeches, and most parameters were statistically significant for the height of Scots pines (*p* > 0.05) (Fig. 2). A multiple regression analysis revealed that tree height was also influenced by diesel oil dose and year of study. The experimental data were best described by the modeled height of Scots pines, where *R*^2^ was determined at 0.92 and standard error of estimation—at 7.6 (Fig. 3).

**Fig. 4:**
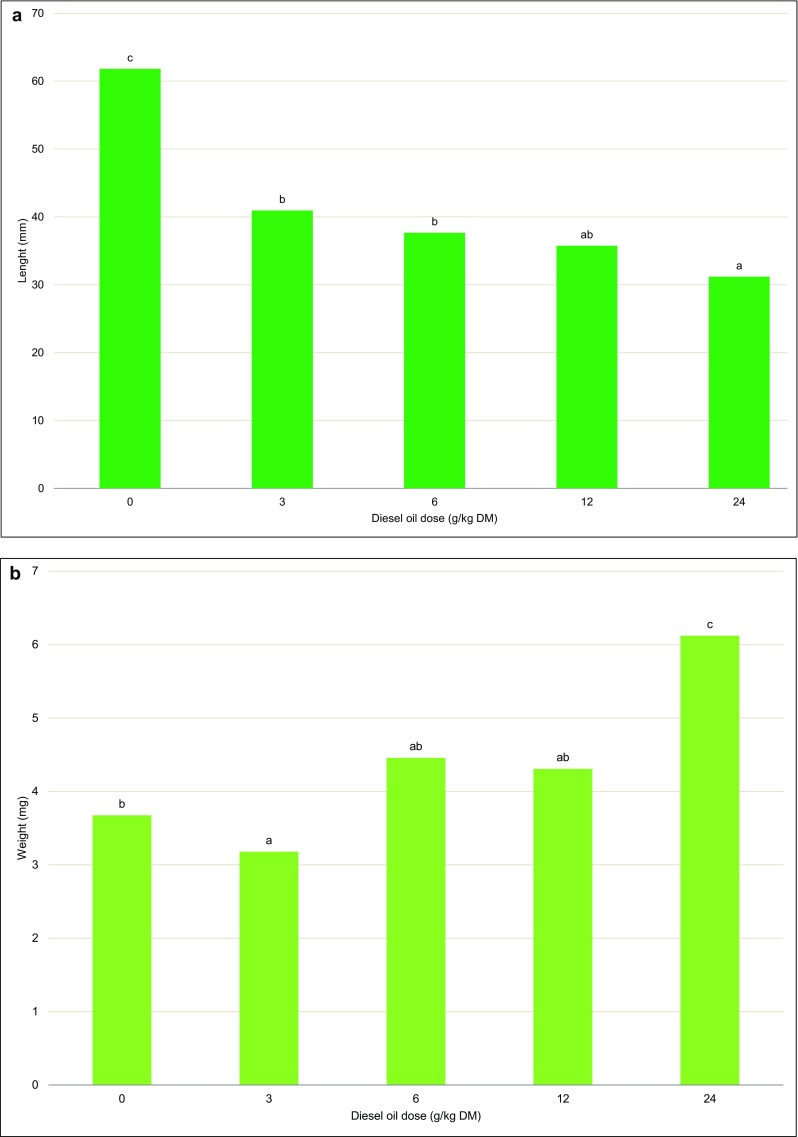
Length (**a**) and weight (**b**) of Scots pine needles. Different letters above the columns indicated significant difference at the *p <* 0.05

Scots pine needles from the control treatment were longest at 61.8 mm on average, whereas the needles from treatments contaminated with the highest doses of diesel oil were twice shorter. Needle weight increased with diesel oil dose. The heaviest needles (6.12 mm) were collected from the treatment with the highest diesel oil dose (24 g/kg soil DM), and they were 66.77% heavier than control needles. Needle thickness also increased with the degree of contamination (Fig. 4). The needles from the most contaminated treatment were 3.3-fold thicker than control needles.

**Fig. 5:**
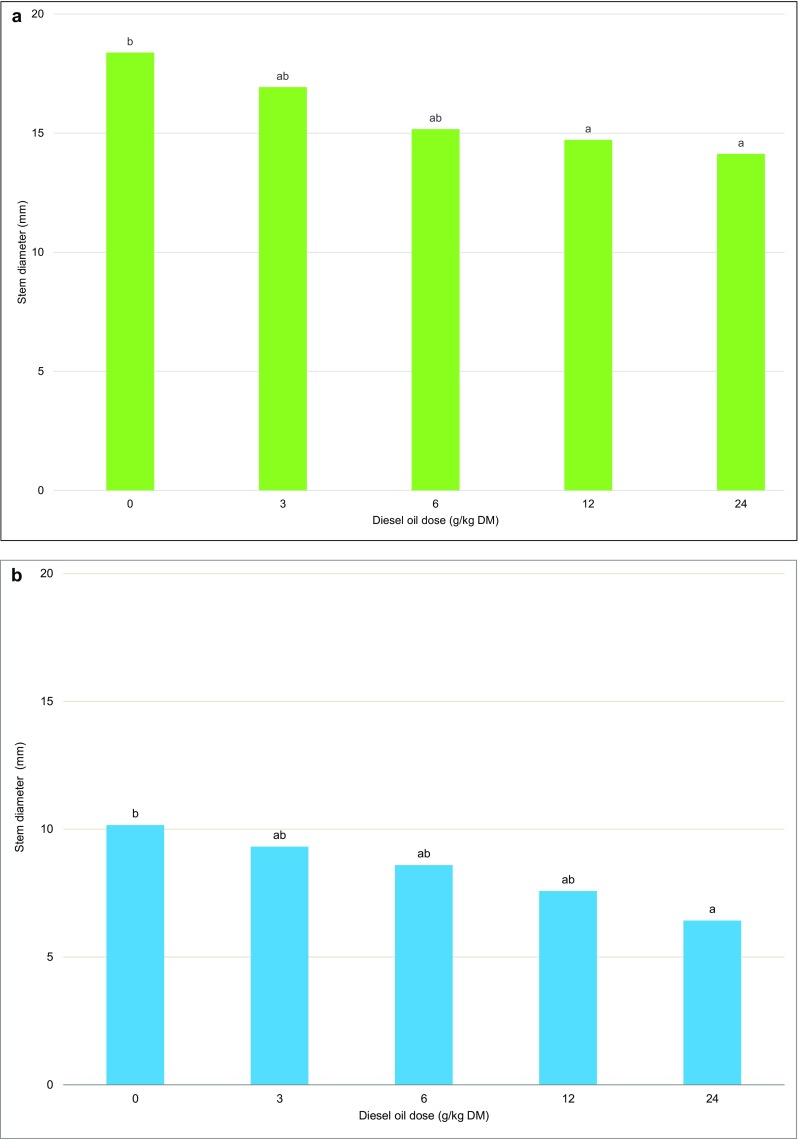
Stem diameter of Scots pine (**a**) and European beech (**b**). Different letters above the columns indicated significant difference at the *p <* 0.05

Scots pines and European beeches growing on soil with the highest concentration of diesel oil were characterized by the smallest stem diameters (Fig. 5). The average stem diameter was nearly twice larger in Scots pines than in European beeches at 15.9 and 8.4 cm, respectively.

**Fig. 6:**
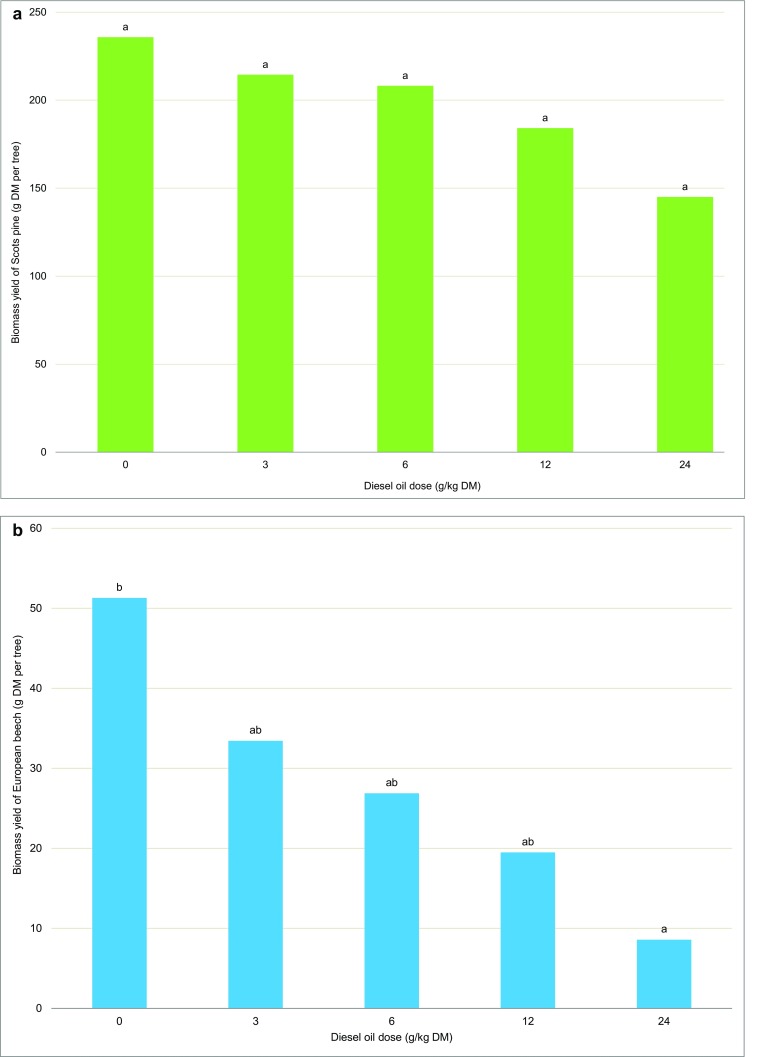
The biomass yield of Scots pine (**a**) and European beech (**b**). Different letters above the columns indicated significant difference at the *p <* 0.05

Unlike in European beeches, the biomass yield of Scots pines was not significantly affected by diesel oil. The biomass yield of European beeches was significantly lower in all contaminated treatments. The analyzed parameter decreased by 83% in the most contaminated treatment, and it decreased by 35%, 37%, and 62% in treatments containing 3, 6, and 12 g of diesel oil/kg of soil DM, respectively (Fig. 6).

### Fluctuating asymmetry

**Fig. 7:**
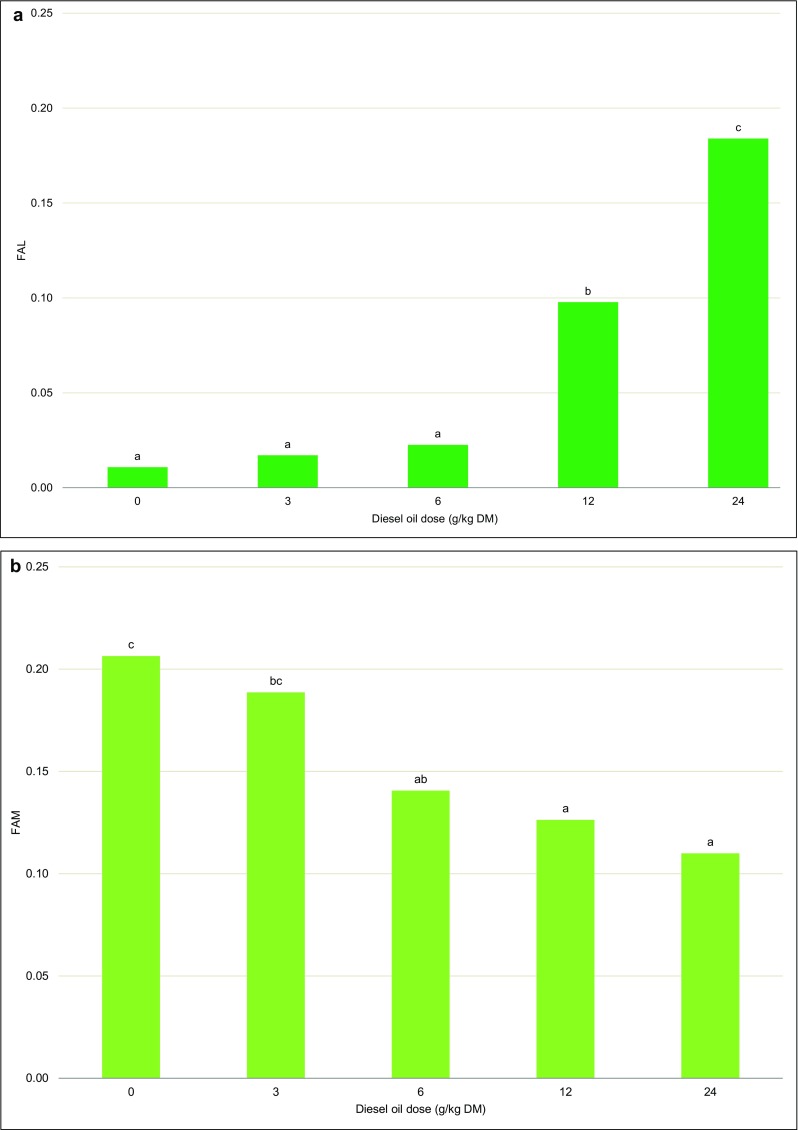
Fluctuating asymmetry indicators—FAL and FAM. Different letters above the columns indicated significant difference at the *p <* 0.05

In the last year of the experiment, fluctuating asymmetry indicators were determined in Scots pine needles before biomass harvest. The analyzed parameters were significantly differentiated by the applied dose of diesel oil (Table 3). The FAL was lowest in the control treatment (0.01). Similar FAL values were noted in Scots pine needles from treatments contaminated with 3 and 6 g of diesel oil/kg of soil DM. The FAL increased significantly with contamination level and was determined at 0.10 and 0.18, respectively (Fig. 7).

**Table 3:** Analysis of variance (*F* test) of fluctuating asymmetry indicators—FAL and FAM

Source of variation	Sum of squares	df	Mean square	*F*	*p* value
FAL					
Intercept	0.3623	1	0.3623	1039.2**	< 0.001
O	0.3130	4	0.0783	224.5**	< 0.001
Error	0.0279	80	0.00033		
FAM					
Intercept	1.9761	1	1.9761	779.7**	< 0.001
O	0.1179	4	0.0295	11.6**	< 0.001
Error	0.2028	80	0.0025		

*O*, diesel oil dose. *Significant at *p* < 0.05; **significant at *p* < 0.01; *ns* not significant

A reverse relationship was observed in the values of FAW. The FAW was highest in the control treatment and in the treatment contaminated with 3 g of diesel oil/kg of soil DM (0.19 and 0.21, respectively), and it was lowest in treatments with higher doses of diesel oil (0.11 to 0.14) (Fig. 7).

### Chlorophyll content, phaeophytization quotient, and leaf greenness index

#### Chlorophyll content

**Fig. 8:**
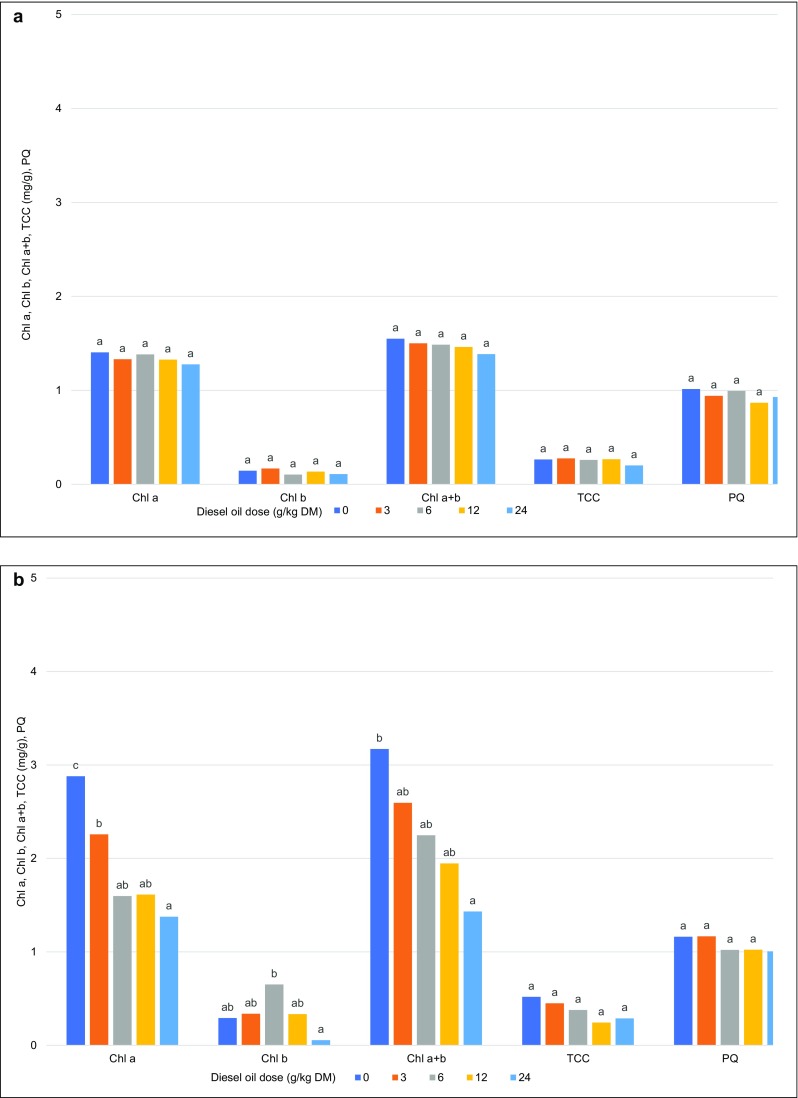

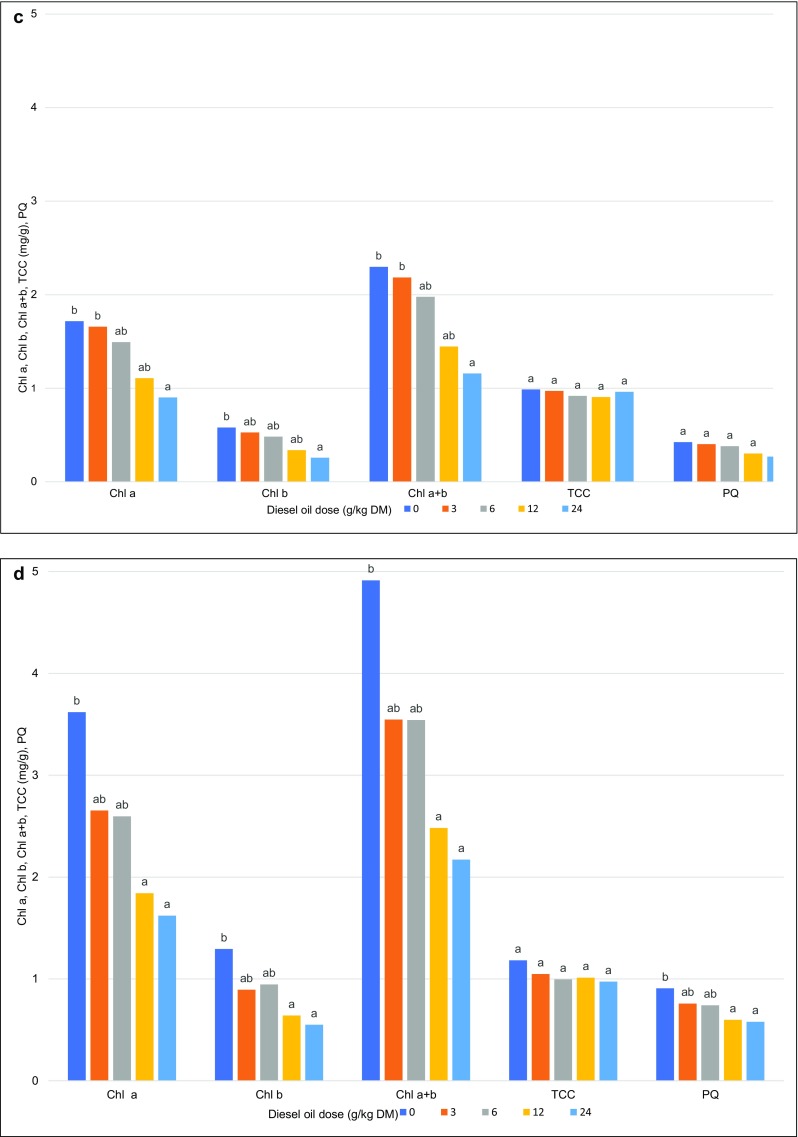
The content of Chla, Chlb, Chla + b, TCC, and PQ and in Scots pine needles (**a** first year, **c** last year) and European beech leaves (**b** first year, **d** last year). Chla, chlorophyll a content; Chlb, chlorophyll b content; Chla + b, the sum of chlorophylls a and b; TCC, total carotenoid content; PQ, phaeophytization quotient. Different letters above the columns indicated significant difference at the *p <* 0.05

In the first year of the study, the content of chlorophylls *a* and *b* and carotenoids in Scots pine needles was not differentiated by the tested doses of diesel oil. In the last year of the study, the concentrations of chlorophylls *a* and *b* in Scots pine needles decreased with a rise in diesel oil dose. In needles collected from the most contaminated treatment, the content of chlorophylls *a* and *b* decreased by 50%. Total chlorophyll content was lowest (1.16) in needles from the treatment contaminated with 24 g of diesel oil/kg of soil DM. In the control treatment, the above parameter was determined at 2.30. Only minor differences in the carotenoid content of needles were observed between treatments (Fig. 8).

In comparison with Scots pine needles, the content of chlorophyll *a* in European beech leaves was higher both in the first and the last year of the experiment. The above parameter decreased significantly with a rise in diesel oil dose. In the last year of the study, the lowest concentration of chlorophyll *a* was noted in leaves from treatments contaminated with 12 and 24 g of diesel oil/kg of soil DM at 1.84 and 1.62 mg/g, respectively. The carotenoid content of European beech leaves was similar to that observed in Scots pine needles (Fig. 8).

#### Phaeophytization quotient

The phaeophytization quotient (PQ) was significantly correlated with the applied dose of diesel oil only in European beech leaves. Control leaves were characterized by higher PQ values than leaves from the most contaminated treatment (0.91 and 0.58, respectively) (Fig. 8).

#### Leaf greenness index

**Fig. 9:**
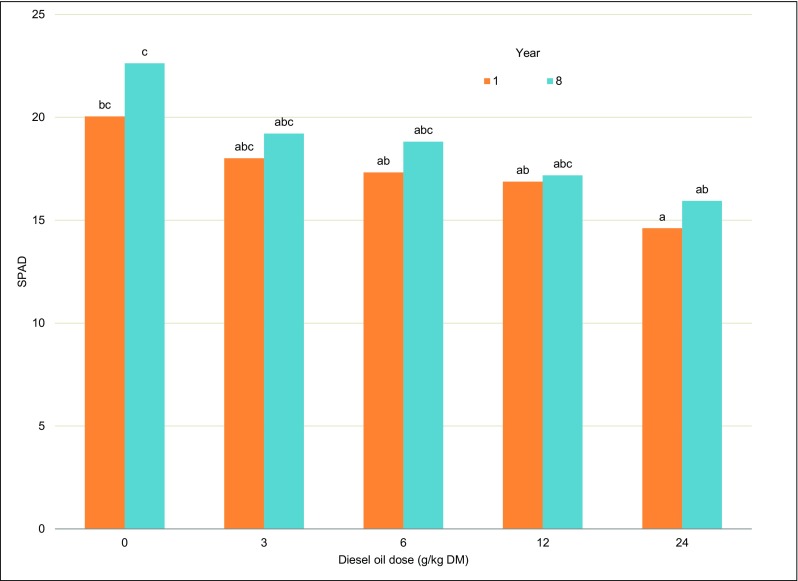
Leaf greenness index (SPAD) of European beech leaves. Different letters above the columns indicated significant difference at the *p <* 0.05

The greenness index of European beech leaves differed significantly between the first and last year of the study and was correlated with soil contamination levels (Fig. 9, Table 4). In the first year of the experiment, the highest SPAD value was noted in control leaves (20.04). The SPAD value of European beech leaves from the most contaminated treatment was 27.0% lower on average (14.61). A similar trend was noted during the second measurement in the last year of the study.

**Table 4:** Analysis of variance (*F* test) of leaf greenness index (SPAD) of European beech leaves

Source of variation	Sum of squares	df	Mean square	*F*	*p* value
Intercept	15,539.0733	1	15,539.0733	3758.2**	< 0.001
O	196.252325	4	49.0630812	11.9**	< 0.001
Y	22.8147655	1	22.8147655	5.5**	< 0.001
O x Y	6.00932462	4	1.50233116	0.4n.s.	0.833
Error	157.120059	38	4.13473839		

*O*, diesel oil dose; *Y*, year; *O* × *Y*, interactions between the factors

*Significant at *p* < 0.05; **significant at *p* < 0.01; *ns* not significant

#### Chlorophyll fluorescence

**Fig. 10:**
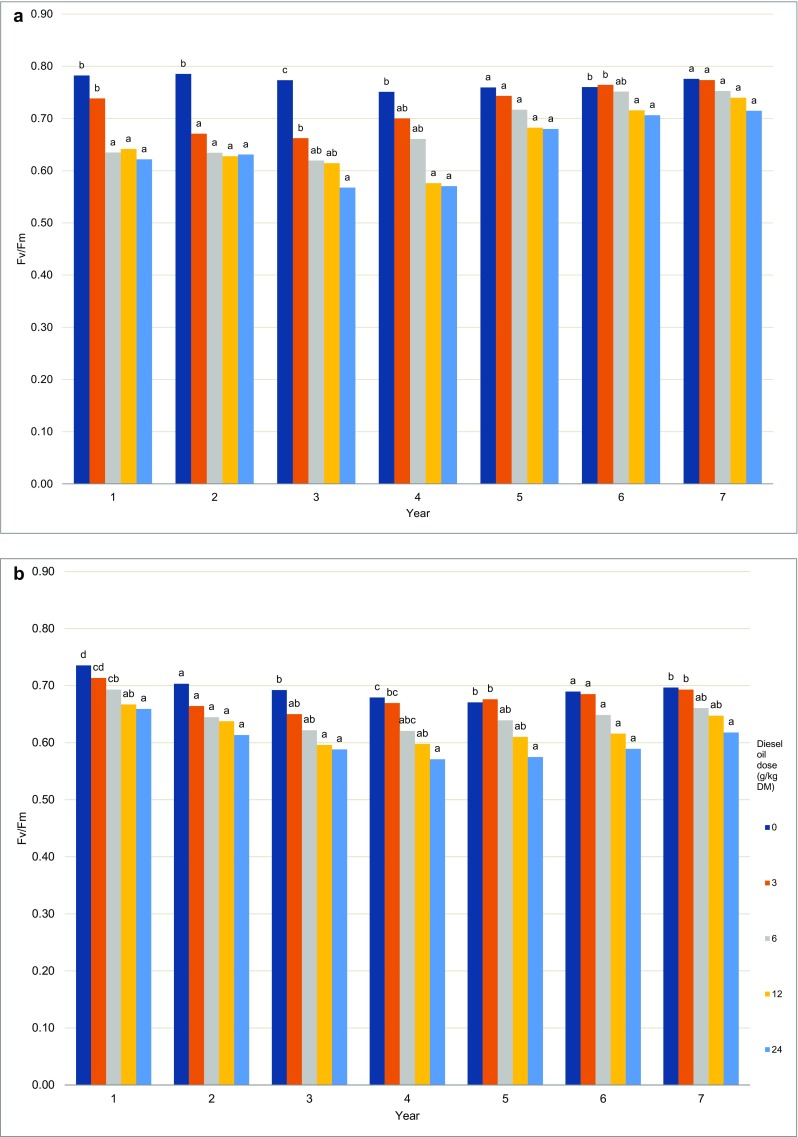
Chlorophyll *a* fluorescence—the Fv/Fm ratio of Scots pines (**a**) and European beech (**b**). Different letters above the columns indicated significant difference at the *p <* 0.05

**Fig. 11:**
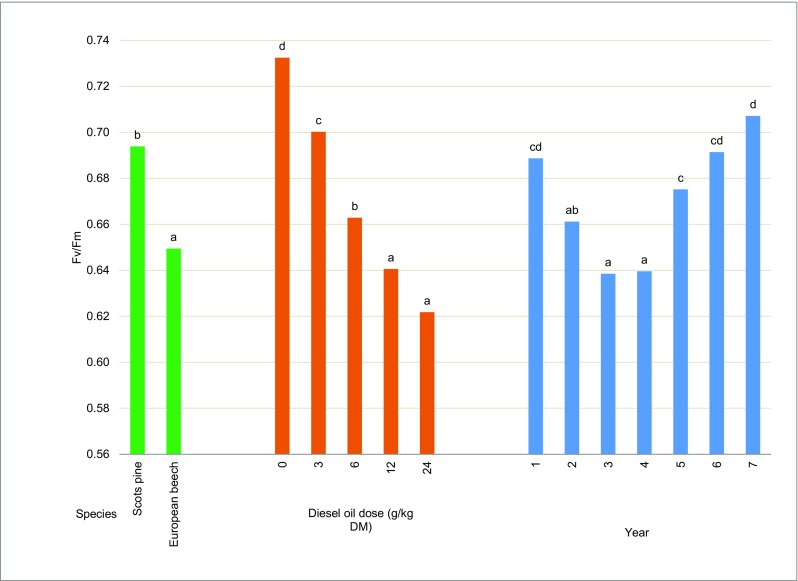
Chlorophyll *a* fluorescence (Fv/Fm ratio) depending on the tree species, the diesel oil dose, and the year of experiment. Different letters above the columns indicated significant difference at the *p <* 0.05

The Fv/Fm ratio of Scots pines growing in all contaminated treatments decreased significantly in the first 4 years of the experiment relative to control. Beginning from year 5, the above parameter increased significantly relative to the first 4 years. The Fv/Fm ratio of control trees ranged from 0.75 to 0.79, and the Fv/Fm ratio of trees grown on the most contaminated soil ranged from 0.57 (in 3 years and 4 years) to 0.71 (in 6 years and 7 years) (Fig. 10). A different trend was observed in European beeches, where the Fv/Fm ratio decreased with an increase in diesel oil dose in all experimental years. The analyzed parameter ranged from 0.67 (in 5 years) to 0.74 (in 1 year) in control plants, and from 0.57 (in 4 years and 5 years) to 0.66 (in 1 year) in the most contaminated treatment (Fig. 11). The results were validated by three-way ANOVA. The Fv/Fm ratio was significantly correlated with tree species, soil contamination level, and experimental year (Table 5). The analyzed parameter was higher in Scots pine needles (0.69) than in European beech leaves (0.65) (Fig. 11). In both tree species, the average Fv/Fm values decreased with increasing soil contamination levels. The analyzed parameter was 18% lower in the most contaminated treatment than in the control treatment (Fig. 11).

**Table 5:** Analysis of variance (*F* test) of chlorophyll *a* fluorescence (Fv/Fm ratio)

Source of variation	Sum of squares	df	Mean square	*F*	*p* value
Intercept	157.4247	1	157.4247	109,654.3**	< 0.001
S	0.1746	1	0.1746	121.6**	< 0.001
O	0.5615	4	0.1404	97.8**	< 0.001
Y	0.2114	6	0.0352	24.5**	< 0.001
S × O	0.0204	4	0.0051	3.5**	< 0.001
S × Y	0.1318	6	0.0220	15.3**	< 0.001
O × Y	0.0776	24	0.0032	2.3**	< 0.001
S × O × Y	0.0478	24	0.0020	1.4n.s.	0.112
Error	0.4005	279	0.0014		

*S*, species; *O*, diesel oil dose; *Y*, year; *S* × *O*, *S* × *Y*, *O* × *Y*, *S* × *O* × *Y*, interactions between the factors

*Significant at *p* < 0.05; **significant at *p* < 0.01; *ns* not significant

## Discussion

The Scots pine is widespread across Northern and Central Europe, and Eastern Siberia. Its geographic range extends westward to Great Britain and Portugal, eastward to Eastern Siberia, southward to the Caucasus, and northward to the Arctic Circle in Scandinavia. The Scots pine is also the most abundant species of coniferous trees in north-eastern Europe (Eckenwalder 2009). In Poland, the species occupies more than 60% of forest area (Central Statistical Office 2017). The Scots pine thrives in nearly all types of habitats, on dry and infertile as well as fertile and moist soils (Muilu-Mäkelä et al. 2015). This pioneer species grows on nutrient-deficient soils, and it is the ideal candidate for soil reclamation, remediation, and reforestation in Central Europe (Pietrzykowski et al. 2013; Placek et al. 2016).

The European beech is one of the main species of forest trees in western and southern Poland. In Europe, its geographic range stretches from the northern Iberian Peninsula through France, Germany, and Central Europe to the Balkans. The European beech is highly tolerant of soil acidification. It thrives on aerated and moist soils (Jarcuska 2009). The European beech is a species of high economic and ecological value, and it is sensitive to drought and spring frost (Pena et al. 2013; Pšidová et al. 2018). The development of European beeches growing on polluted soil for many years has never been described in the literature.

Petroleum and petroleum derivatives are the most ubiquitous pollutants in highly industrialized countries. Grasses, legumes, and trees (phytoremediation) are used to remove or neutralize these pollutants in the environment (soil and water). Phytoremediation can pose an alternative to other purification techniques because it does not modify soil properties (Cook and Hesterberg 2013; Burezq and Aliewi 2018). Phytoremediation methods rely on plant species that rapidly adapt to degraded environments and are capable of producing large quantities of biomass (Cook and Hesterberg 2013; Dudai et al. 2018). The Scots pine is one of such species (Placek et al. 2016), whereas the use of European beech for phytoremediation has not been described in the literature.

Diesel oil decreases the biomass yield of plants, and its effects are exacerbated with an increase in soil pollution (Leewis et al. 2013; Xi et al. 2018; Xie et al. 2018; Petrová et al. 2017; Bamgbose and Anderson 2015; Villacís et al. 2016). In our study, increasing doses of diesel oil reduced the biomass yield of European beech only. European beech was characterized by the lowest yield in the most contaminated treatment. The biomass yield of Scots pine was not significantly correlated with an increase in diesel oil dose, and it was more than 700% higher in comparison with European beech. The absence of significant differences in the yield of Scots pine grown on polluted soil for 8 years indicates that this species was more resistant to contamination than the European beech (Fig. 6). Other authors also demonstrated that the Scots pine well adapts to polluted environments. Scots pines growing in a former mining region for 30 years were characterized by higher yields than control trees (Pietrzykowski and Socha 2011). In southern Finland, where the Scots pine is the predominant tree species, this conifer was also resistant to chemical stress resulting from soil contamination with lead (Selonen and Setälä 2015). However, the biomass yield of Scots pine grown for 10 years on flotation sediments in south-west and mid-west Poland was reduced by 50% (Mleczek et al. 2018).

The biomass yield of trees is determined mainly by biometric parameters such as tree height and stem diameter. In this experiment, Scots pines were taller and had thicker stems than European beeches after 8 years (Fig. 1; 5). Kuznetsova et al. (2010) also observed that Scots pines were taller than deciduous trees in degraded habitats. In the first and second year of the present experiment, Scots pines did not respond to contamination, and variations in height were observed from year 3. In years 7 and 8, significant differences in tree height were not observed, which could indicate that the Scots pine adapted to contaminated soil. In contrast, European beeches responded to diesel oil pollution already in the first year of the study. Significant differences in the height of European beeches exposed to different doses of diesel oil were observed throughout the experiment (Fig. 2). Palmroth et al. (2002) analyzed 1-year-old seedlings of Scots pine and eastern cottonwood on soil contaminated with diesel oil (at a concentration of 0.5% and 2.0%). Poplar leaves were damaged in the treatment polluted with 0.5% diesel oil, and some seedlings did not survive in the treatment contaminated with 2.0% diesel oil. The Scots pine was more resistant to oil pollution. Needles were not damaged, and the length and annual increase in needle length were identical to those noted in control trees. In the first year of the experiment, the average increment in tree height was determined at 8 cm in Scots pines and 4.3 cm in European beeches (Fig. 4). The above suggests that the initial height increment influenced the growth performance of trees in the course of the 8-year-long experiment. Similar observations were made by Villacís et al. (2016). Diesel oil pollution also impaired the growth of plants in the tropical climate (Merkl et al. 2005; Shirdam et al. 2008).

The stem diameter in the analyzed tree species was significantly differentiated by the applied dose of diesel oil. In the most contaminated treatment, the stem diameter of Scots pines was 1.5-fold lower, and the stem diameter of European beeches was more than twice smaller than in the control treatment (Fig. 5). Acacia seedlings growing on soil contaminated with diesel oil from a leaking underground pipe in the arid region of Arava in southern Israel were also characterized by a reduced stem diameter (Tran et al. 2018). In contrast, the stem diameter of five out of more than 20 tree species growing in oil fields in the Amazon drainage basin in Ecuador was greater than in the control species (Villacís et al. 2016).

The length of Scots pine needles from the most contaminated treatment was 50% smaller relative to control needles. However, the shortest needles were also heaviest (Fig. 4). The length, thickness, surface area, fresh and dry weight, and the number of leaf stomata per unit area are generally determined by light intensity (Kyć 1981). In north-eastern China, the anatomical, morphological, and physiological traits of leaves in shrubs, perennial grasses, and forbs were affected by differences in the utilization of water resources (Guo et al. 2017). High leaf thickness enables plants to maintain relatively high leaf water content during drought (Marenco et al. 2009; Seelig et al. 2015). Needle length in 6-week-old Scots pine seedlings growing on substrates with various zinc concentrations also decreased with a rise in zinc concentration (Ivanov et al. 2016). The needles of 1-year-old Scots pine seedlings growing on soil contaminated with diesel oil (0.5% and 2.0%) were not damaged, and the annual increment in needle length was similar to that noted in control needles (Palmroth et al. 2002). For this reason, Scots pine needles are often used in research as bioindicators of environmental contamination from various sources (Pająk et al. 2017).

In ecological studies, the FA values of leaves and needles are often analyzed as stress indicators (Fair and Breshears 2005; Cuevas-Reyes et al. 2018; Zvereva et al. 1997). The FA values of Scots pine needles exposed to radioactive elements (Geraskin et al. 2003; Kashparova et al. 2018), heavy metals (Kozlov and Niemelä 1999), air pollution (Kozlov et al. 2002), and water stress (Kozlov and Niemelä 2003) have been described in the literature. The fluctuating asymmetry index increases in response to stress, which was also confirmed in our study (Fig. 7). The FAL values of Scots pine needles were highest in the most contaminated treatments (12 and 24 g of diesel oil/kg of soil DM). A reverse correlation was noted in the FAM values of needles which were highest in the least contaminated treatment (Fig. 7). Our results suggest that Scots pines growing on polluted soil for 8 years developed various adaptive strategies. Water accumulation in cells could be one of the strategies for minimizing the adverse effects of diesel oil. Plants rely on three common strategies for drought adaptation: escape, tolerance, and avoidance (Chaves et al. 2003). Soil contamination with high xenobiotic concentrations leads to physiological drought (Baciak et al. 2017). Excessive levels of sodium chloride in soil decrease osmotic potential and inhibit water uptake by plants (Wang et al. 2011; Yilmaz 2007; Sikorski et al. 2013).

The chlorophyll content of plants is one of the key indicators of growth, photosynthetic ability, productivity, and stress tolerance. Chlorophyll concentrations decrease under exposure to stressors such as gas pollution (Lange et al. 2004; Nevalainen et al. 2014; Sakugawa and Cape 2007), oxidative stress (Biczak 2016), and heavy metals (Costa et al. 2018; Xiong et al. 2018). In our experiment, diesel oil decreased the concentrations of chlorophylls *a* and *b* (Fig. 8). Plant growth was probably inhibited by chlorophyll degradation during its biosynthesis. Diesel oil can suppress chlorophyll synthesis, which decreases the content of chlorophylls *a* and *b* (Wang et al. 2011). The concentrations of photosynthetic pigments were also reduced in Scots pines exposed to air pollution (Joshi and Swami 2009).

The content of chlorophyll *a* in Scots pine needles was measured in the first (2 months after soil contamination) and last year of the study (before biomass harvest). The concentrations of chlorophylls *a* and *b*, carotenoids, total chlorophyll, and the phaeophytization quotient remained unchanged during the first measurement (Fig. 8). The above suggests that the synthesis of photosynthetic pigments was not inhibited in plants growing on polluted soil for only 2 months. In contrast, the content of assimilation pigments in Scots pine needles and European beech leaves was correlated with the applied xenobiotic dose after 8 years of exposure. Chlorophyll content was lowest in Scots pine needles grown in the most contaminated treatment. Control needles and needles from the least contaminated treatment (3 g/kg) were characterized by comparable chlorophyll levels of 1.72 and 1.66 mg/g FW, respectively. Similar results were reported by Wang et al. (2011) in soil containing the smallest doses of diesel oil (1, 5, and 10 g/kg). The chlorophyll content of reed plants increased, which suggests that the analyzed species was tolerant of diesel oil, in particular at lower concentrations. However, the content of total chlorophyll and chlorophylls *a* and *b* decreased in treatments where contamination levels exceeded 15 g/kg. The observed responses to low contamination levels indicate that plants rely on diesel oil as a source of components for the synthesis of selected enzymes (Wang et al. 2011). In our study, the content of chlorophyll *a* in Scots pine needles from all contaminated treatments was 68% higher than the content of chlorophyll *b*. In needles collected from the most contaminated treatment, the concentration of chlorophyll *b* was 45% lower than in control needles. The differences in carotenoid content were not statistically significant. Total chlorophyll content was similar to the concentration of chlorophyll *a* (Fig. 8).

The European beeches are angiosperms, whereas Scots pines are gymnosperms. The leaves of the analyzed tree species differ in anatomy and morphology, but chlorophyll synthesis, initiation of chloroplast development, and the expression of genes encoding light-harvesting chlorophylls *a*/*b* are determined by light exposure in both species (Chinn and Silverthorne 1993; Sarijeva et al. 2007). European beech leaves differ in sensitivity to UV-B subject to habitat conditions (natural and experimental) (Láposi et al. 2009), whereas forest seedlings are better adapted for lower levels of UV-B radiation than older trees (Grant et al. 2005). Sarijeva et al. (2007) analyzed leaf and chloroplast adaptation to light and shade and concluded that European beech was more resistant to both insolation and shade than *Ginkgo biloba* L.

European beech leaves contained more chlorophylls *a* and *b* than Scots pine needles, both in the first (difference of 35%) and the last year of the study (45%). Chlorophyll *a* content was lowest in leaves from treatments contaminated with the two highest doses of diesel oil (49% in the first year, and 55% in the last year of the experiment).

The chlorophyll content of *Accacia raddiana* leaves growing on soil contaminated with diesel oil was reduced by 70% (Tran et al. 2018). In our study, the concentrations of carotenoids and chlorophylls *a* and *b* in European beech leaves also decreased with increasing diesel oil doses, both in the first and the last year of the experiment. Carotenoid content decreased by 44% and 18%, and total chlorophyll content was reduced by 50% and 55% in the first and last year of the study, respectively. The content of photosynthetic pigments in *Mimosa caesalpiniaefolia*, *Erythrina speciosa*, and *Schizolobium parahyba* also decreased under exposure to lead (Ribeiro de Souza et al. 2012).

Similarly to chlorophyll *a* content, the greenness index of European beech leaves decreased with a rise in diesel oil dose. The greenness index of alfalfa and bristle grass growing on oil-contaminated soil also decreased in a study by Xie et al. (2018). The greenness index and chlorophyll *a* content of European beech leaves increased after 8 years of exposure to diesel oil (Fig. 9). The above suggests that the health status of European beeches improved in the last year of the experiment.

Chlorophyll fluorescence is a reliable indicator of plant stress, but it does not support the identification of different stress types. Fluorescence measurements are performed to evaluate the overall physiological status of plants before the first symptoms of leaf damage become apparent. Fluorescence intensity changes in response to the duration and intensity of stress (Dąbrowski et al. 2016; Kalaji et al. 2018). The Fv/Fm ratio is a reliable indicator of the photochemical activity of the photosynthetic apparatus. The above parameter measures the efficiency of light utilization in the initial stages of photosynthesis, and its value is proportional to the maximum quantum yield of PSII (Baker et al. 2001; Lawson et al. 2002). In healthy leaves, the maximum quantum yield of PSII (Fv/Fm) ranges from 0.76 to 0.85 (Percival et al. 2006). A decrease in the value of the above parameter testifies to plant stress. Very low values of Fv/Fm (0.2–0.3) are indicative of irreversible changes in the structure of PSII (Banks 2018; Kalaji et al. 2018; Percival et al. 2006; Sakugawa and Cape 2007). Rapid fluorescence measurements conducted with the use of various techniques indicate that environmental stressors induce specific photosynthetic responses in trees (Pšidová et al. 2018).

The Fv/Fm ratio is the most popular measure of chlorophyll fluorescence (Papageorgiou 2010). Two related ratios, Fv/Fm and Fv/Fo, and the decrease in Chl fluorescence are often used to determine the potential quantum yield of PSII (Lichtenthaler and Babani 2004). Photosynthetic efficiency expressed by the Fv/Fm ratio was determined at 0.7 in healthy pine needles growing under field conditions (Salmela et al. 2011). In Scots pine needles fumigated with nitrous acid (HONO), the Fv/Fm ratio decreased significantly relative to control needles (Sakugawa and Cape 2007). In our experiment, the Fv/Fm ratio of Scots pine needles decreased with a rise in soil contamination in the first, second, third, and fourth year of the study. However, the differences in the Fv/Fm values of needles from treatments contaminated with different doses of diesel oil were less pronounced in the fifth, sixth, and seventh year of the experiment. In the last year, the Fv/Fm ratio of needles ranged from 0.7 to 0.8, which indicates that the trees were in good condition.

The Scots pine was characterized by higher values of Fv/Fm, which could suggest that this species was more resistant to increasing levels of pollution than the European beech (Fig. 10). The European beech responded to contamination already in the first year of the study, and a minor decrease in Fv/Fm values was observed in successive years. However, the health status of European beeches did not improve to the extent noted in Scots pines. The morphological and physiological parameters of European beeches were compromised under exposure to increasing concentrations of diesel oil. A decrease in Fv/Fm values was observed in water-stressed European beeches and maples (Pšidová et al. 2018; Banks 2018) and in the leaves of the Pará rubber tree infected with fungi (Sterling and Melgarejo 2018). A study of selected tree and shrub species, including *Betula pendula* Roth, *Sorbus intermedia* Ehrh., *Physocarpus opulifolius* [L.] Maxim., *Spiraea japonica* L., and *Hedera helix* L., exposed to high levels of particulate matter pollution in downtown Warsaw revealed a decrease in Fv/Fm values of with a rise in contamination levels. The Fv/Fm ratio decreased in all evaluated plants, but *Hedera helix* L. was the most sensitive species (Popek et al. 2018). The maximum quantum yield of PSII (Fv/Fm) also decreased in eucalyptus trees exposed to Cd (Pietrini et al. 2015). The Fv/Fm ratio of willows (*Salix fragilis* (Sf) and *Salix aurita* (Sa)) decreased after 100 days of exposure to metals (Zn, Cu, Cd, Ni) relative to control (Evlard et al. 2014).

Plants respond differently to the presence of toxic substances in the environment, and phytotoxicity results from the interactions between chemical compounds and plants growing under specific environmental conditions. Chemical compounds cause morphological deformations and induce quantitative and qualitative changes in various plant organs (Tran et al. 2018; Wang et al. 2011; Sikorski et al. 2013).

## Conclusions

Our findings confirmed the hypothesis that soil contamination with diesel oil induces various changes in the morphological and physiological properties of European beeches and Scots pines.

The Scots pine better adapts (grows more rapidly and produces higher biomass) to long-term soil contamination with diesel oil than the European beech. The results of this study suggest that the Scots pine is a potential candidate species for soil phytoremediation.

In European beeches, growth inhibition and leaf discoloration (a decrease in chlorophyll content) were observed already after the first year of the experiment, which indicates that 1-year-old seedlings of European beech are robust bioindicators of soil contamination with diesel oil.
